# People, Professionals, and Profit Centers: The Connection between Lawyer Well-Being and Employer Values

**DOI:** 10.3390/bs12060177

**Published:** 2022-06-03

**Authors:** Patrick R. Krill, Nikki Degeneffe, Kelly Ochocki, Justin J. Anker

**Affiliations:** 1 Krill Strategies, LLC, Minneapolis, MN 55408, USA; 2 Department of Psychiatry and Behavioral Sciences, University of Minnesota, Minneapolis, MN 55455, USA; degen022@umn.edu (N.D.); kellyochocki2@gmail.com (K.O.); anke0022@umn.edu (J.J.A.)

**Keywords:** wellbeing, stress, feedback, lawyers, mental health

## Abstract

Concerns about the well-being of lawyers are rising against the backdrop of a transforming legal profession, one which many observe to be operating more like a business in recent decades. However, aspects of this change, such as lawyers perceiving that their employers value financial performance and productivity above all else, could be associated with unhealthy work practices detrimental to lawyer well-being. The objective of the present study was to determine whether the perceived values of employers were differentially associated with lawyer well-being, stress, and work overcommitment. To this end, 1959 participants from a random sample of attorneys completed a survey designed to assess well-being. Participants were separated into one of three groups based on what they perceived their employer to value most about them: (1) Professionalism/Individual (professionalism and skills), (2) Financial Worth/Availability (revenue generation and availability), and (3) No Value/No Feedback (feeling unvalued or lacking feedback) and compared on measures of mental and physical health (SF-12), stress (Perceived Stress Scale), and work over commitment (Effort–Reward Imbalance Questionnaire). MANOVA results indicated that mental health, stress, and work overcommitment significantly differed between groups in the following rank order: Professionalism/Individual > Financial Worth/Availability > No Value/No Feedback. Overall, our findings paint a compelling picture of a health hierarchy within legal work environments, one that appears to be linked to employer values.

## 1. Introduction

“Money is at the root of virtually everything that lawyers don’t like about their profession: the long hours, the commercialization, the tremendous pressure to attract and retain clients, the fiercely competitive marketplace, the lack of collegiality and loyalty among partners, the poor public image of the profession, and even the lack of civility. Almost every one of these problems would be eliminated or at least substantially reduced if lawyers were simply willing to make less money.” —Patrick J. Schiltz, “On Being a Happy, Healthy, and Ethical Member of an Unhappy, Unhealthy, and Unethical Profession”.

[1]

The proposition that money underlies many of the legal profession’s challenges is not new. The widely cited quote from Schiltz’s 1999 law review article reflects a decades-long transition underway in the legal profession, one that has seen the pursuit of profits become the top priority [2]. On a related note, many legal scholars have observed that law has become more of a business than a profession, with both law firm prestige and individual career success often tied to profits and money [3].

However, while the financial performance of law firms has risen, growing empirical evidence suggests the mental well-being of members of the legal profession has fallen. For example, findings from a nationwide study of over 12,000 lawyers indicated that the rate of substance use and mental health problems among lawyers significantly exceeds the rate in the general population [4]. In addition, a recent study of over 2800 randomly sampled California and Washington, D.C., lawyers demonstrated that high levels of mental health and substance use problems were associated with work overcommitment and work–family conflict, especially among women [5]. Similar findings have been demonstrated internationally, including a large 2021 study that described a global crisis in lawyer mental well-being, stating that no one jurisdiction or section of the profession is unaffected. According to that research, key issues contributing to difficulties with mental well-being include the stressful nature of the work, intensive work/time demands, poor work–life balance, and high levels of pressure [6]. 

Studies from other fields undergoing a similar profit-centric transformation support a connection between increased financial performance and decreased employee well-being. For example, a systematic review of 50 studies in the nursing home industry concluded that the field’s for-profit expansion has resulted in worse employee well-being [7]. Often, even well-intentioned efforts to promote well-being in an environment driven by profits face significant hurdles. In what is described as a “performance-health paradox”, aspirational, health-oriented management practices (e.g., providing sufficient buffers, latitudes, and resources to employees to reduce stress and promote adequate recovery from work) typically collide with the demands of a profit-centered organization. The resulting tradeoff between economic performance and employee health manifests as greater job demands and increasing workload to the detriment of employee well-being [8]. 

Several studies have demonstrated that high job demands contribute to poor mental health [9]. For example, in one study of 60,556 fulltime workers, the number of hours an employee perceived they were expected to work was the number one predictor of symptom severity of depression, anxiety, and other mental health problems [10]. Job stress and long work hours are also associated with a heightened risk of physical illnesses such as cardiovascular disease [11,12]. Indeed, a recent study by the World Health Organization indicated that people working 55 or more hours each week face an estimated 35% higher risk of a stroke and a 17% higher risk of dying from heart disease compared to people following the widely accepted standard of working 35 to 40 h in a week [13]. Moreover, a meta-analysis of 79 studies reporting cross-sectional and longitudinal relationships between physical symptoms and various occupational stressors indicated that workplace stressors were significantly related to numerous physical symptoms, including backache, headache, eyestrain, sleep disturbance, dizziness, fatigue, appetite, and gastrointestinal problems [14]. Poor mental and physical health stemming from job stress also poses a financial risk for employers. Some estimates suggest that job stress costs U.S. employers more than USD 300 billion annually and may cause 120,000 excess deaths each year [15]. 

Beyond the stress and pressure brought about by a focus on profits, the role of employer feedback in employee mental and physical health is also critical. Workplace stressors may be increased by a failure to provide feedback, which may signal to employees that they are not an integral part of the organization and that their work is not essential, thus undermining their well-being [16]. Conversely, supportive workplaces where people feel valued are closely linked to employee happiness and well-being [17]. Healthy and happy employees have a better quality of life, a lower risk of disease and injury, increased work productivity, and a greater likelihood of contributing to their communities than employees with poorer well-being [18].

Despite the increasing commercialization of the legal profession and the rising mental health issues among lawyers appearing to occur in tandem, the relationship between the two phenomena has yet to be systematically examined. As such, this study aims to address this knowledge gap by examining the relationship between the perceived values of employers and critical aspects of individual employee well-being, including stress, physical health, and mental health. 

Since the goal of the present study was to determine the relationship between lawyer mental health and well-being and the perceived values of employers, we placed participants into “value groups” demonstrative of and consistent with workplaces that evince either a profession-centric or business-centric approach to the practice of law. A third group consisted of lawyers who felt unvalued by their employer or who lacked insight into what their employer valued about them. Finally, we hypothesized that these “value groups” would differ based on measures of health (stress, mental health, and physical health) and the presence of maladaptive workplace practices (e.g., overcommitment and permissiveness toward alcohol in the workplace). Based on these anticipated differences, we further hypothesized that a focus on productivity and financial contributions would be associated with worse health. 

## 2. Materials and Methods

### 2.1. Participants

#### Recruitment and Random Selection

The study design and protocol were reviewed by the University of Minnesota Institutional Review Board and deemed exempt from approval. The recruitment and methods for survey distribution are described in detail in Anker and Krill [5]. Briefly put, participants were randomly selected from a list of unique deidentified IDs supplied by the California Lawyers Association (CLA) and D.C. Bar to receive an email containing a link to our survey. Clicking on the link directed participants to the informed consent page of the survey. The study was conducted from May to June of 2020. As our interest was to assess perceived employer value, the sample was restricted to lawyers who were employed part- or fulltime in legal settings with a managerial-based structure such as a private practice law firm, corporate inhouse legal department, government agency, or public interest or nonprofit practice setting. Solo practitioners were excluded from analyses. The final sample consisted of 1959 participants who had complete data on the study measures. 

### 2.2. Perceived Employer Value/Value Groups

Three groups were formed based on participants’ response to the following item, “What do you feel your employer values most about you?” The three groups were as follows: (1) Professionalism/Individual—value in skill, professionalism, and human worth (e.g., “My overall talent and skill as a lawyer”), (2) Financial Worth/Availability—value in terms of employee’s availability and ability to produce revenue (e.g., “My productivity or hours I bill”), and (3) No Value/No Feedback—perceives employer does not value them or provides little feedback (e.g., “I don’t know—I get very little feedback”). Table 1 lists the specific items associated with each value group and the participant response frequency of each item. 

## 3. Materials

### 3.1. Descriptive Variables

Demographics and work-related variables. Information regarding gender, age, race, relationship status, and lifetime diagnosis of a mental health disorder were collected and reported for each group. Additionally, information on the following work-related variables were collected from participants: average number of hours worked per week, position (e.g., Managing Partner, Senior Partner, Junior Partner, etc.), and law practice setting (e.g., private firm, government, corporate, etc.). 

### 3.2. Outcome Measures 

Perceived Stress Scale. The 10-item Perceived Stress Scale (PSS) is a widely used psychometrically reliable measure of the degree to which situations in one’s life are appraised as stressful [19]. Items on the PSS are designed to tap into how unpredictable, uncontrollable, and overloaded respondents find their lives. Responses are on a 5-point Likert scale with the following options: 0 = Never, 1 = Almost never, 2 = Sometimes, 3 = Fairly often, and 4 = Very often (score range: 0 to 40). Sample items include “In the last month, how often have you been upset because something happened unexpectedly?” and “… how often have you found that you could not cope with all the things you had to do?”

Mental and Physical Health. The SF-12 Health Survey was used to assess physical and mental health within the sample [20]. Items in the SF-12 are designed to measure health concepts, such as ability to function physically, body pain, role limitations due to physical health and emotional problems, general mental health, and ability to function socially. Sample questions include “During the past four weeks, how much did pain interfere with your normal work (including both work outside the home and housework)?” and “During the past four weeks, how much of the time has your physical health or emotional problems interfered with your social activities (like visiting with friends, relatives, etc.)?” As documented in the SF-12 user manual, a norm-based scoring algorithm was used to derive a general physical health score and a general mental health score. This algorithm allowed for scores within this study to be compared with scores in the general U.S. population. Scores above 50 in the present study indicated better physical or mental health than the general population, whereas scores below 50 suggested poorer physical or mental health than the general population. 

Work Overcommitment. The Work Overcommitment subscale of the Effort–Reward Imbalance Questionnaire [21] assesses the extent to which respondents feel overwhelmed by their work demands. The subscale consists of five items that measure overcommitment on a 4-point Likert scale (1 = Strongly Disagree, 2 = Disagree, 3 = Agree, 4 = Strongly Agree). Example items from the questionnaire include “As soon as I get up in the morning, I start thinking about work problems,” “Work rarely lets me go; it is still on my mind when I go to bed,” and “I get easily overwhelmed by time pressures at work.” Scores on the Overcommitment scale range from 6 (low overcommitment) to 24 (high overcommitment).

Workplace Permissiveness Toward Alcohol. Five items from the Your Workplace (YWP) questionnaire were used to assess the frequency of activities cueing alcohol consumption within the vocational environment (e.g., going drinking with coworkers after work or talking about drinking during work hours) [22]. The following is an example item from the subscale: “In some jobs, you’re not supposed to drink during working hours, or on breaks or at lunchtime, but some employees drink anyway. How many times in the past six months have your friends at work done this?” Scores on the Support for Consumption scale were calculated using the scoring algorithm supplied by Beattie et al. [22] and ranged from 9 (low support for consumption) to 36 (high support for consumption). 

Author-Generated Questions. Four items (see Table 4) were created by the authors to assess general perceptions about the connection between workplace behaviors, mental health, and substance use in the legal profession.

### 3.3. Statistical Analyses

Sociodemographic and work characteristics (descriptive measures) were compared between groups using Pearson Chi-Square tests for categorical data and one-way between-subjects analysis of variance (ANOVA) for continuous measures. Our primary objective was to examine the extent to which mental and physical health, stress, workplace alcohol permissiveness, and work overcommitment relate to lawyers’ beliefs about what their employer values most about them. To test this, group comparisons were performed with a between-subjects multivariate analysis of variance (MANOVA) to account for multiple correlated outcomes of the group membership variables. Group differences were examined on five measures: SF-12 Mental Health Composite score, SF-12 Physical Health Composite score, total score of the Perceived Stress Scale, Work Overcommitment score, and Workplace Permissiveness Toward Alcohol. To test for potential covariates, gender, age, and lifetime diagnosis of a mental health disorder were also included. Additionally, since the survey occurred during the COVID-19 pandemic, an additional covariate was included that assessed the perceived influence of COVID on mental health. Significance level was set at <0.05 and statistical analyses were conducted using Statistical Package for Social Sciences version 26 (IBM, Armonk, NY, USA). 

## 4. Results

### 4.1. Sociodemographics

Women comprised approximately 50% (*n* = 970) of the sample. Table 2 shows the frequency of other demographics for each group. The Professionalism/Individual group consisted of a greater proportion of men (52% of this group were male) compared to the Financial Worth/Availability group (46% were male), while the opposite was true for women—the Professionalism/Individual group was made up of 47% women and the Financial Worth/Availability group was made up of 53% women. Lawyers in the youngest age cohort (age 30 or younger) made up 16% of the Financial Worth/Availability group, whereas the youngest cohort only made up 7% of the Professionalism/Individual group and 8% of the No Value/No Feedback group. In contrast, lawyers in the oldest age cohort (61 or older) made up 21% of the Professionalism/Individual group and only 10% of the Financial Worth/Availability group. Regarding race, lawyers that identified as nonwhite were more likely to indicate that their employer did not value them or did not provide feedback. With respect to relationship status, 72% in the Professionalism/Individual group were married, compared to 61% in the Financial Worth/Availability group. Additionally, a greater proportion of lawyers in the No Value/No Feedback group were divorced compared to the Professionalism/Individual group (14% vs. 8%). Regarding self-reported diagnoses, 48% of lawyers in the No Value/No Feedback groups reported a lifetime diagnosis of a mental health disorder, while 41% in the Financial Worth/Availability group and 38% of lawyers in the Professionalism/Individual group reported a mental health disorder.

### 4.2. Work-Related Demographics

Work-related sample demographics are shown in Table 3. Regarding the number of hours worked in a typical week, a significantly greater proportion of the Financial Worth/Availability group worked 51 h or more (37%) compared to the Professionalism/Individual group (24%). Concerning position, lawyers in the Professionalism/Individual group tended to be in more senior positions relative to the other two groups. Finally, lawyers in the Financial Worth/Availability group were significantly more likely to work in private practice and significantly less likely to work in a government setting compared to the other two groups. 

### 4.3. Legal Profession and Mental Health

Table 4 shows the frequency of participants within each group who responded “yes,” “no,” or “unsure” to items related to perceptions that the legal profession has contributed to maladaptive behaviors, poor mental health, and drinking/substance use and whether they have contemplated leaving due to job-related burnout or stress. Relative to lawyers in the Professionalism/Individual value group, lawyers in the Financial Worth/Availability and No Value/No Feedback group were significantly more likely to perceive their workplace facilitating maladaptive behaviors. Similarly, relative to lawyers in the Professionalism/Individual group, lawyers in the Financial Worth/Availability and No Value/No Feedback group were significantly more likely to report the legal profession had been detrimental to their mental health. In fact, nearly 50% of those in the No Value/No Feedback group and 41% in the Financial Worth/Availability group selected “yes” to this item, compared to 24% in the Professionalism/Individual group A significantly greater proportion of the Financial Worth/Availability group (vs. the Professionalism/Individual group) indicated their time in the legal profession caused their alcohol or drug use to increase. Finally, in response to the question, “Are you considering, or have you left the profession due to mental health, burnout, or stress?” 37% of lawyers in the No Value/No Feedback group, 27% of the Financial Worth/Availability group, and 15% of the Professionalism/Individual group selected “Yes”. 

### 4.4. MANOVA Results

Table 5 presents the means, standard deviations, and results of the MANOVA for all continuous outcome measures for the sample and by group. Using an alpha level of 0.01 to evaluate homogeneity assumptions, Box’s M test of homogeneity of covariance (*p* = 0.35) and Levene’s homogeneity test (all *p*’s ≥ 0.05) were not statistically significant, confirming equality of variance between groups. Results from the preliminary MANOVA model indicated that the participants’ gender (Wilks’ Lambda A = 0.985, F(5, 1780) = 5.369, *p* < 0.001, ƞ^2^ = 0.015), age (Wilks’ Lambda A = 0.853, F(5, 1780) = 61.30, *p* < 0.001, ƞ^2^ = 0.147), lifetime mental health diagnosis (Wilks’ Lambda A = 0.90, F(5, 1780) = 41.56, *p* < 0.001, ƞ^2^ = 0.105), and effect of COVID on health (Wilks’ Lambda A = 0.99, F(5, 1780) = 5.42, *p* < 0.001, ƞ^2^ = 0.015) were significantly associated with the outcome measures and were therefore included in the final model as covariates. The final model demonstrated a significant multivariate effect for the three groups on the primary outcome measures (Wilks’ Lambda A = 0.941, F(10, 3560) = 11.03, *p* < 0.001, ƞ^2^ = 0.03), meaning that the three groups differed in a statistically meaningful way with respect to the outcome measures (while accounting for covariates and correlations between the outcome measures). 

Separate univariate analyses of between-subject effects were used to examine group differences with respect to each outcome measure. It is important to note that these results do not account for correlations between outcomes but rather pertain to each outcome alone. Univariate results indicated that groups significantly differed with respect to PSS (*F*(2, 1883) = 54.78, *p* < 0.000); SF-12 physical health composite (*F*(2, 1928) = 5.17, *p* = 0.006); SF-12 mental health composite (*F*(2, 1982) = 45.90, *p* < 0.000); your workplace (*F*(2, 1791) = 2.94, *p* = 0.053); and work overcommitment (*F*(2, 1900) = 8.54, *p* < 0.000).

### 4.5. Discriminate Analysis Results

The MANOVA was followed up with discriminant analysis to examine the linear combinations in more detail. The resulting discriminate function identified the unique combinations of outcome variables (variate/functions) that best differentiated groups and provided information on how specific outcome variables contribute to variate combinations. To account for uneven group sizes in our sample, prior probabilities was determined based on observed group size. 

The analysis revealed two discriminate functions. The first explained 79.5% of the variance, canonical R^2^ = 0.059, whereas the second explained 20.5%, canonical R^2^ = 0.01. In combination, these discriminant functions significantly differentiated the groups, A = 0.926, X^2^ (10) = 138.34, *p* < 0.000, and removing the first function indicated that the second function remained a significant group differentiator, A = 0.984, X^2^ (4) = 28.883, *p* = 0.00. Thus, both variates have an important and unique impact on the model given their high explanatory power (model accuracy), indicating that group differences can be explained in terms of two underlying dimensions of relationships between groups and the outcome variables. 

**Figure 1:**
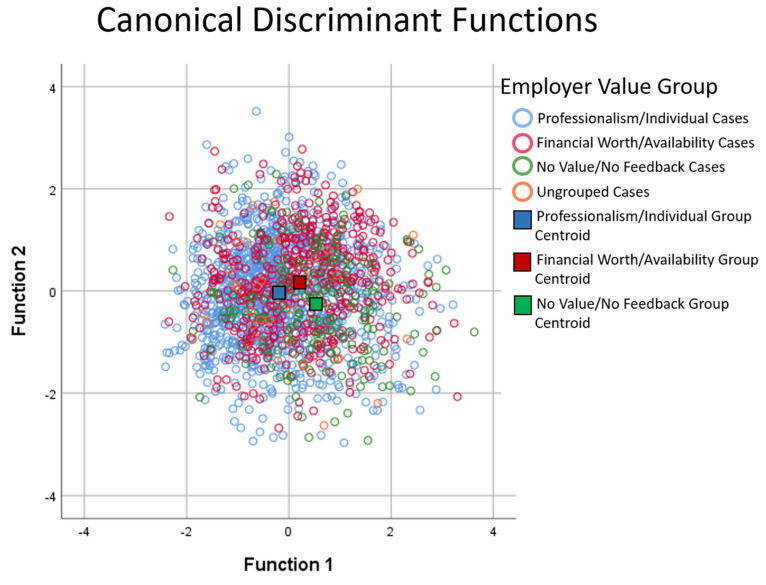
Canonical discriminant function graph showing discrimination between the three employer value groups (Professionalism/Individual; Financial Worth/Availability; No Value/No Feedback).

To explore the nature of these relationships and to identify which variables/variable combinations are most important to differentiating groups, within-group correlations between the discriminating variables (outcome measures) and standardized canonical discriminant function coefficients were calculated. The structure matrix values provide information on the relative contribution of each variable to the variates. The resulting correlations revealed that perceived stress/PSS (r = 0.93) and SF12-mental health (r = −0.79) loaded highly on the first function, Your Workplace score (r = 0.69) and, to a lesser extent, SF12-physical health (r = 0.38) loaded on the second function, and Work Overcommitment loaded heavily on both functions (Function 1: r= 0.68 and Function 2: r = 0.61). To better visualize these group distinctions, employer value group centroids were plotted on a discriminant function plot. As shown in Figure 1, Function 1, consisting of the PSS, SF12-mental health, and Work Overcommitment variables, effectively discriminated all groups (see horizontal separation between group centroids), while Function 2, consisting of the Your Workplace score, SF12 Physical Health, and Work Overcommitment, discriminated the No Value/No Feedback group from the other groups (see vertical separation between group centroids). Thus, differences between all employer value groups are largely due to differences in the PSS, SF12-Mental Health, and Work Overcommitment scores while the Your Workplace, Work Overcommitment, and SF12-Physical Health scores more effectively differentiated the No Value/No Feedback group from the other two value groups, who were comparable with respect to these measures. 

## 5. Discussion

Our research offers both good and bad news for the legal profession, along with many instructive findings that lend themselves to the formulation of concrete strategies for improving the mental health of lawyers. Beginning with the good, a majority of lawyers (62%) belonged to the Professionalism/Individual group and thus reported feeling most valued by their employer for things that can reasonably be characterized as positive, such as important professional skills and attributes or inherent worth as a human being. Across several key domains that we examined, lawyers in the Professionalism/Individual group fared significantly better than their peers in the other two groups in terms of personal well-being. 

Regarding perceived stress, mental health, and work overcommitment, a discernible trend emerged between our three groups, resulting in what might be described as a health hierarchy. Specifically, lawyers in the Professionalism/Individual group reported better mental health, with lawyers in the Financial Worth/Availability group reporting worse outcomes. The group with the worst health and most limitations are those who either felt unvalued by their employer or did not have enough feedback to know what their employer values most about them (the “No Value/No Feedback” group). Those in the No Value/No Feedback group experienced worrisome levels of perceived stress that would clearly warrant employer intervention due to their likely association with mental health problems among their lawyers. 

Based on previous reports within the legal profession, we would hope and expect that most lawyers would indeed find themselves in the Individual/Professional grouping. For example, a recent survey of competency expectations for associate development indicates that many law firms expect their associate lawyers to develop skills in three general areas: traditional legal and communication skills, character traits and relationship skills, and a client-focused orientation [23]. Similarly, a large, multiyear survey of lawyers throughout the U.S. revealed that most believe that character traits such as integrity, trustworthiness, and conscientiousness are of primary importance for lawyers to succeed early in their careers, more so than their ability to generate business [24]. 

Although productivity is not typically or expressly identified as a competency, it may nonetheless be implied by the fact that billable hours are generally part of most performance reviews in law firms. Indeed, 27% of lawyers reported that their employer values their productivity, availability, or ability to generate revenue the most (the “Financial Worth/Availability group”). This finding would seem to mark a disconnect from what many law firms and lawyers publicly report as being important markers of development and success. Finally, approximately 10% of lawyers reported feeling unvalued at work or not knowing what their employer values most about them. Combined, these 37% of lawyers who are not part of the Individual/Professional group are experiencing the worst health. 

Overall, these findings align with prior research outside of law, which has found that employees who feel valued are more likely to report better physical and mental health as well as higher levels of engagement, satisfaction, and motivation compared to those who do not feel valued by their employers [18,25,26]. Given the established impact of feeling valued on engagement and motivation, as well as its relationship with mental and physical health that we uncovered with this research, it is paradoxical that legal employers who value productivity and financial contributions above professional skill and human worth may be experiencing both lower levels of productivity and higher healthcare costs. 

Law firms may be quick to dismiss the suggestion that they are experiencing high costs associated with lost productivity when their lawyers are outwardly meeting billable hour requirements and thus performing at a high level. They would be mistaken to do so, because our findings clearly suggest that lawyers in the Financial Worth/Availability group experienced worse health than their counterparts in the Professionalism/Individual group. This is perhaps unsurprising, recalling the performance–health paradox, which suggests that the productivity demands of a profit-focused organization often prevail over any efforts to support employee health [8]. However, when lawyers experience ill-health, they are presumptively delivering lower-quality work and doing so less efficiently, even while meeting their billable hour obligations. After all, when people are sick, they are distracted by their ailments and have trouble concentrating. This may ultimately result in client dissatisfaction with work product and loss of future business opportunities for the employer. Furthermore, some research has shown that costs associated with a lack of productivity among unhealthy employees were even higher than the direct medical claims costs associated with sick workers [15]. 

Stress and decreased well-being can also result in diminished cognitive function in lawyers [27], which presents other risks such as an increased likelihood of costly mistakes, problems which are on the rise even as many law firms are reporting record profits. In fact, payouts for legal malpractice claims reached an all-time high in 2020 [28]. Additionally, legal employers with an unhealthy workforce are more likely to experience significant costs associated with high attrition. Our data revealed that more than one-third of lawyers reported feeling valued most for their productivity or availability or were a part of the No Value/No feedback group. Consequently, those lawyers were experiencing worse health and were significantly more likely to report that their time in the legal profession had been detrimental to their mental health and caused their use of alcohol or drugs to increase. They were also, by a large margin, more likely to report contemplating leaving the legal profession due to mental health, burnout, or stress. These findings present meaningful economic risk for legal employers. It has been estimated that unwanted associate attrition costs a law firm with 100 associates USD 5.6 million annually and a firm with 500 associates USD 28 million annually [29]. When a more experienced lawyer or partner in a law firm leaves, the costs can be exponentially higher. Given the potentially significant financial stakes involved, it would seem clear that legal employers have compelling incentives to examine whether they are valuing the right things about their lawyers and, if so, whether they are effectively communicating those values. 

Legal employers who can make their lawyers feel more valued for their skill or humanity rather than their productivity and responsiveness may be able to improve their lawyers’ well-being and simultaneously mitigate unwanted turnover, both of which may be even more pressing aims for legal employers following the COVID-19 pandemic. Prior to the pandemic, data suggested that attrition rates were about 10 times higher in law firms than they are in well-run corporations, with an ultimate price tag well over USD 1 billion dollars each year for the top 200 law firms alone [30]. During COVID-19, turnover intentions for many lawyers appear to have increased due to rising stress, work overcommitment, and work–family conflict. Indeed, recent research conducted during the pandemic revealed that more than 20% of lawyers contemplated leaving the legal profession due to mental health, burnout, or stress [5]. 

Employers who make their lawyers feel valued for their skill and human worth may also be able to reduce their overall healthcare costs, which will likely be a growing priority given the increasing propensity for ill-health present in younger Americans more generally, especially in the aftermath of the COVID-19 pandemic. Recent studies suggest that one-third of millennials in the general population have health conditions that reduce their quality of life and life expectancy [31]. They also have substantially higher diagnoses for eight of the top ten health conditions than the preceding generation, and based on their current health status, millennials are more likely to be less healthy when they are older compared to prior generations. As such, the prospect of significantly increased medical expenses would appear to be looming for legal employers, which underscores the importance and value of addressing management practices or aspects of firm culture that may be contributing to ill-health now. Being proactive in this regard is essential, as research has shown that, with relatively few exceptions, once people are in a high-risk health category and develop a chronic disease, it is unlikely that they will move back into a low-risk category [15]. In other words, prevention is the most cost-effective approach to reducing healthcare expenses. 

Outside of what they value most about their lawyers, our research offers at least a partial roadmap for how employers may begin to redress other aspects of their organizational culture that may also be precipitating stress and poor mental health. Specifically, we asked respondents whether their workplace fosters, rewards, or normalizes maladaptive behaviors. Lawyers in the Financial Worth/Availability or No Value/No Feedback groups were more than twice as likely to answer yes, thereby providing additional evidence of another layer of dysfunction that may exist in those employment settings. While we did not specifically define maladaptive behaviors, there are well-known categories of such conduct that have been documented by prior research. For example, bullying and sexual harassment have recently been shown to be rife in the legal profession [32], incivility appears to be on the rise, with 85% of lawyers having experienced uncivil or unprofessional behavior in the last 6 months [33], and hazardous drinking is widespread [34]. By targeting and seeking to improve such problem behaviors in their workplace, employers may be able to improve the stress levels and mental health of their lawyers.

Type of employment setting was also implicated in our findings. Specifically, lawyers working in private firms were significantly less likely to feel valued for their skill or human worth and far more likely to report feeling most valued for their productivity. This finding is perhaps unsurprising given that law firms are obviously more focused on revenue generation than corporate legal departments or government agencies. Lawyers working in corporations were most likely to be part of the Professionalism/Individual group, while lawyers working in government were most likely to be part of the No Value/No Feedback group. If we combine the Financial Worth/Availability group and the No Value/No Feedback group together, however, we see that the biggest proportion of this group, by a wide margin, is made up of private firm lawyers. This indicates that, as a cohort, private firm lawyers experience the worst mental and physical health.

Furthermore, within private firms overall, we found additional stratification based on firm size. Prior research indicates that large-firm lawyers have a lower probability of good health and a higher probability of poor health relative to those in the public sector and those in solo practices and small firms [35]. Similarly, our findings indicated that the larger the firm, the less likely lawyers are to feel valued for their professional or human worth, and the more likely they are to feel most valued for their financial and productivity contributions and, consequently, report worse health. While it would be tempting under such circumstances to assign responsibility for lawyer ill-health solely to the employer, the values of lawyers themselves cannot be ignored. Reports from the field suggest those values appear conflicted and often inconsistent. For example, recent industry surveys suggest that millennial lawyers are becoming increasingly open to leaving their current firm, with dissatisfaction with work–life balance being the number one reason why. In the same survey, however, respondents indicated that they value a firm’s compensation package over all other factors when evaluating potential employers. This was a change from prior surveys indicating that respondents primarily valued work–life balance [36]. 

These conflicting values also echo the performance–health paradox, which manifests at an individual level in contradictory goals related to performance and goal achievement versus need for recovery to protect personal health and opportunities to pursue nonwork interests [8]. Importantly, a gender divide appears to exist on this issue, with more male respondents signaling that compensation was most important and more female lawyers prioritizing work–life balance. Such a gender divide might be expected considering research showing that women in the legal profession experience higher levels of perceived stress, depression, anxiety, and hazardous drinking than men and are more likely to leave the profession due to work–family conflict [5]. 

Prior research has shown that workplace permissiveness toward alcohol use is a primary predictor of risky drinking among men and women in the legal profession, thus supporting the perception of an alcohol-based social culture that has long typified the legal profession [5]. Given that risky and hazardous drinking are longstanding and widespread challenges for the profession, we sought to understand whether perceived employer values had any bearing on workplace permissiveness toward alcohol use. There did not appear to be a relationship between these phenomena, perhaps suggesting that the legal profession’s drinking norms and cultural embrace of alcohol are of a more deeply seated and systemic nature that transcends employer values. 

Turning to the bad news, we found that lawyers are in poor health overall. The general health of lawyers, as measured by SF-12, falls below the general population. This is true irrespective of which of our three categories lawyers fall into regarding what their employer values most about them. In sum, although working in a legal employment environment that makes lawyers feel valued most for their professionalism or human worth translates into better mental and physical health than working in a legal employment environment that does not, a lawyer’s health is still likely to be worse than that of a member of the general population. This striking finding takes on additional significance because lawyers tend to fall higher on the socioeconomic scale, and it is typically people of lower socioeconomic status who are more likely to have worse self-reported health and lower life expectancy and suffer from more chronic conditions when compared with those of higher socioeconomic status [37].

## 6. Limitations

Results should be interpreted with consideration of the study’s limitations. First, we did not assess what individual lawyers valued most about being a lawyer. It is reasonable to assume that employer/employee alignment on the value placed on generating revenue would be associated with better, not worse, mental health. Future research on perceived value would benefit from assessing the extent to which alignment (or misalignment) of employer–employee value systems are associated with the health and well-being of practicing lawyers. Results from such a study could shed light on the importance of tailoring employer feedback to better support the value of their employees.

Second, the cross-sectional design of the study precludes determination of cause-and-effect relationships between perceived employer value and the health and work-related measures assessed in the study. While there would be obvious ethical concerns with directly manipulating what an employer values most about their employees, a systematic investigation of lawyer health before and after the implementation of a program that coaches employers on how to effectively communicate employee value could shed light on such cause-and-effect relationships. 

Third, it is possible that preexisting mental health conditions may have biased some participants to perceive that their employer did not value them. To reduce this likelihood, past mental health diagnosis was controlled for in our analysis, but it is still possible that unaccounted-for conditions or symptoms may have influenced the perceptions of some participants. 

## 7. Conclusions

From upholding democracy and the rule of law to safeguarding individual freedoms and ensuring the orderly operation of economies and institutions, lawyers have an indispensable job to do. As such, increased visibility into the causes of their ill-health holds significant utility. Overall, our findings paint a compelling picture of a health hierarchy within legal work environments, one that appears linked to the apparent value systems of employers as well as their ability to effectively communicate those values through the provision of adequate feedback. Based on our findings, our hypothesis that a business-centric approach to practicing law has the potential to negatively impact the health and well-being of lawyers appears to be confirmed. Lawyers who work in environments that value professionalism, skill, and humanity over productivity and availability are in better health and experience lower levels of stress than their counterparts in other work environments. Future research in this area may add valuable nuance to the broader findings that a primary focus on productivity is associated with worse health among lawyers.

Furthermore, the importance of providing clear and regular feedback is obvious from our findings since the lawyers reporting the highest levels of stress and worst mental health are those who either feel unvalued or do not know what their employer values most about them. Employers would be well-served in heeding the lessons contained in these novel and actionable findings. Recognizing and seeking to disrupt self-defeating management practices—such as valuing productivity above skill, talent, and human worth, or failing to provide meaningful feedback and make employees feel valued—would be wise pursuits for employers seeking to both improve the lives of their employees and strengthen the organization’s financial performance. For individual lawyers themselves, better understanding the relationship between their own health and well-being and what their employer values most about them should hopefully allow for more informed decisions about the type of work environment they choose.

## Figures and Tables

**Table 1:** Perceived employer value items and participant response frequency to the question, “What do you feel your employer values most about you?”.

	Individual	
	N	%
**Professionalism/Individual Items**		
“My overall talent and skill as a lawyer”	566	28.9%
“Everything, they value my inherent worth as a human being”	470	24.0%
“My leadership abilities”	65	3.3%
“My professionalism and ethics”	48	2.5%
“My interpersonal or communication skills”	37	1.9%
“My intellectual and academic contributions to the profession”	35	1.8%
*Total N*	*1222*	*62.4%*
**Financial Worth/Availability Items**		
“My productivity or the hours I bill”	361	18.4%
“My responsiveness and availability”	130	6.6%
“My ability to generate business”	48	2.5%
*Total N*	*539*	*27.5%*
**No Value/No Feedback Items**		
“I don’t know—I get very little feedback”	132	6.7%
“Not much—my employer does not make me feel valued”	66	3.4%
*Total N*	*198*	*10.1%*

**Table 2:** Sociodemographic variables.

	Professionalism/Individual		Financial Worth/Availability		No Value/No Feedback		Chi-Square	*p*-Value
	N	%	N	%	N	%		
**Gender**							9.60	0.04
Women	572	46.9% ^a^	288	53.4% ^b^	110	55.6% ^a,b^		
Men	638	52.3% ^a^	248	46.0%^b^	86	43.4% ^a,b^		
*Total N*	*1221*		*539*		*198*			
**Age**							61.36	0.000
≤30	86	7.0% ^a^	87	16.1% ^b^	15	7.6% ^a^		
31–40	337	27.6% ^a^	170	31.5% ^a^	62	31.3% ^a^		
41–50	290	23.8% ^a^	115	21.3% ^a^	45	22.7% ^a^		
51–60	253	20.7% ^a^	111	20.6% ^a^	46	23.2% ^a^		
61 or older	255	20.9% ^a^	56	10.4% ^b^	30	15.2% ^a,b^		
*Total N*	*1221*		*539*		*198*			
**Race**							26.53	0.001
Asian or Pacific Islander	82	6.7% ^a^	33	6.2% ^a^	19	9.6% ^a^		
Black/African American	54	4.4% ^a^	24	4.5% ^a, b^	18	9.1% ^b^		
Caucasian/White	1010	82.9% ^a^	429	80.5% ^a^	140	70.7% ^b^		
Latino/Hispanic	36	3.0% ^a^	26	4.9% ^a^	10	5.1% ^a^		
Native American	3	0.2% ^a^	0	0.0% ^a^	0	0.0% ^a^		
More than one race or Other	22	1.8% ^a^	14	2.6% ^a, b^	9	4.5% ^b^		
*Total N*	*1218*		*533*		*198*			
**Relationship Status**							42.08	0.000
Married	877	71.8% ^a^	327	60.8% ^b^	127	64.8% ^a,b^		
Divorced, Separated, or Widowed	98	8.0% ^a^	43	8.0% ^a,b^	27	13.8% ^b^		
Single, with significant other	109	8.9% ^a,b^	61	11.3% ^b^	9	4.6% ^a^		
Single, never married	137	11.2% ^a^	107	19.9% ^b^	33	16.8% ^a,b^		
*Total N*	1221		538		196			
**Diagnosis of Mental Health Disorder**							7.25	0.027
	466	38.1% ^a^	221	41.0% ^a, b^	95	48.0% ^b^		

Within each row, each superscript letter denotes column proportions that did not differ significantly at the 0.05 level according to Pearson Chi-Square tests.

**Table 3:** Work-related demographics.

	Professionalism/Individual		Financial Worth/Availability		No Value/No Feedback		Chi-Square	*p*-Value
	N	%	N	%	N	%		
**Hours worked in a typical week**							33.33	0.000
≤30 h	83	6.8% ^a^	22	4.1% ^a^	13	6.6% ^a^		
31 to 40 h	266	21.8% ^a^	90	16.8% ^b^	50	25.3% ^a^		
41 to 50 h	573	47.0% ^a^	228	42.5% ^a^	81	40.9% ^a^		
≥51 h	298	24.4% ^a^	196	36.6% ^b^	54	27.3% ^a, b^		
*Total N*	*1220*		*536*		*198*			
**Position in Legal Profession**							103.92	0.000
Managing partner	158	12.9% ^a^	21	3.9% ^b^	24	12.1% ^a^		
Senior partner	245	20.0% ^a^	76	14.1% ^b^	28	14.1% ^a,b^		
Junior partner	95	7.8% ^a^	50	9.3% ^a^	13	6.6% ^a^		
Of counsel	124	10.1% ^a^	61	11.3% ^a^	16	8.1% ^a^		
Senior associate	219	17.9% ^a,b^	106	19.7% ^b^	23	11.6% ^a^		
Junior associate	119	9.7% ^a^	123	22.8% ^b^	33	16.7% ^b^		
Other	262	21.4% ^a^	102	18.9% ^a^	61	30.8% ^b^		
*Total N*	*1222*		*539*		*198*			
**Employer Type**							41.35	0.000
Private	694	56.8% ^a^	371	68.8% ^b^	102	51.5% ^a^		
Government	377	30.9% ^a^	103	19.1% ^b^	66	33.3% ^a^		
Corporate	136	11.1% ^a^	49	9.1% ^a^	26	13.1% ^a^		
*Total N*	*1222*		*539*		*198*			

Within each row, each superscript letter denotes column proportions that did not differ significantly at the 0.05 level according to Pearson Chi-Square tests.

**Table 4:** Legal profession and mental health.

	Professionalism/Individual		Financial Worth/Availability		No Value/No Feedback		Chi-Square	*p*-Value
	N	%	N	%	N	%		
**Does your workplace foster, reward, or normalize maladaptive behaviors?**							183.54	0.000
Yes	117	9.6% ^a^	126	23.4% ^b^	56	28.3% ^b^		
No	757	62.1% ^a^	188	34.9% ^b^	50	25.3% ^c^		
Unsure	345	28.3% ^a^	224	41.6% ^b^	92	46.5% ^b^		
*Total N*	*1219*		*538*		*198*			
**Has your time in the legal profession been detrimental to your mental health?**							88.54	0.000
Yes	293	24.0% ^a^	221	41.1% ^b^	93	47.2% ^b^		
No	696	57.1% ^a^	212	39.4% ^b^	66	33.5% ^b^		
Unsure	230	18.9% ^a^	105	19.5% ^a^	38	19.3% ^a^		
*Total N*	*1219*		*538*		*197*			
**Has your time in the legal profession caused your use of alcohol and/or other drugs to increase?**							20.63	0.000
Yes	157	12.9% ^a^	112	20.8% ^b^	31	15.7% ^a,b^		
No	972	79.7% ^a^	383	71.1% ^b^	147	74.2% ^a,b^		
Unsure	91	7.5% ^a^	44	8.2% ^a^	20	10.1% ^a^		
*Total N*	*1220*		*539*		*198*			
**Are you considering leaving, or have you left, the profession due to mental health, burnout, or stress?**							80.95	0.000
Yes	188	15.4% ^a^	144	26.7% ^b^	74	37.4% ^c^		
No	970	79.6% ^a^	354	65.7% ^b^	106	53.5% ^c^		
Unsure	61	5.0% ^a^	41	7.6% ^a^	18	9.1% ^a^		
*Total N*	*1219*		*539*		*198*			

Within each row, each subscript letter denotes column proportions that did not differ significantly at the 0.05 level according to Pearson Chi-Square tests.

**Table 5:** Means and MANOVA results for continuous measures.

	Professionalism/Individual	Financial Worth/Availability	No Value/No Feedback	*p* Value	Partial ƞ^2^
Perceived Stress Scale	14.79 (6.92)	17.43 (7.04)	19.34 (7.13)	<0.000	0.041
SF-12 Physical Health (<50 = below national norms)	46.16 (5.69)	46.15 (6.07)	44.81 (6.59)	0.015	0.005
SF-12 Mental Health (<50 = below national norms)	46.71 (10.25)	43.17 (10.61)	41.22 (10.99)	<0.000	0.026
Your Workplace	18.85 (5.54)	19.82 (5.48)	18.30 (5.95)	0.053	0.003
Work Overcommitment	14.43 (3.72)	15.94 (3.91)	15.74 (3.68)	<0.000	0.024

## Data Availability

Data cannot be shared publicly because they involve human research participants and contain potentially sensitive information related to mental health and substance use. Researchers who meet the criteria for access to confidential data may request to access the data by contacting the corresponding author and completing a University of Minnesota Data Use Agreement.
